# Atypical Small Acinar Proliferation: Utility of Additional Sections and Immunohistochemical Analysis of Prostatic Needle Biopsies

**DOI:** 10.5812/numonthly.2067

**Published:** 2012-03-01

**Authors:** Julián Arista-Nasr, Omar Martínez-Mijangos, Braulio Martínez-Benítez, Leticia Bornstein-Quevedo, Saul Lino-Silva, Shaddaí Urbina-Ramírez

**Affiliations:** 1 Department of Pathology, Instituto Nacional de Ciencias Médica y la Nutrición, S. Z. (INCMNSZ), Tlalpan, México D.F, México; 2 Departament of Pathology, Instituto Nacional de Cancerología (INCan), México D. F, México

**Keywords:** Prostate, Biopsy, Needle, Carcinoma, Neoplasm, Residual

## Abstract

**Background:**

In surgical pathology, atypical small acinar proliferation is commonly detected in prostate biopsies. Most studies on atypical small acinar proliferation have examined morphological characteristics and the utility of immunohistochemical studies. However, these resources are not available to many pathology departments. We have found that examining additional sections is a simple and inexpensive method that allows better evaluation of focal prostatic glandular atypia.

**Objectives:**

The present report compares the diagnostic utility of immunohistochemical techniques versus examining additional sections in prostate biopsies with focal glandular atypia.

**Patients and Methods:**

Thirty recently studied prostate biopsies with focal glandular atypia were selected. In each case, 3 additional levels were examined. An immunohistochemical study was performed on one level using an antibody against high-molecular-weight keratin (34BetaE12). Two additional sections were stained with hematoxylin and eosin.

**Results:**

The diagnosis of focal carcinoma was established with only additional sections in 4 cases (13.3%). In 2 of these biopsies, additional areas of carcinoma were found that were not identified in the original sections. In 4 other cases, immunohistochemical analysis was the only useful method for diagnosing cancer. In 9 cases (30%), both methods were useful for classifying focal glandular atypia as carcinoma. In the remaining 13 cases,neither immunohistochemical analysis nor additional sections were useful in changing the diagnosis of focal glandular atypia.

**Conclusions:**

Focal glandular atypia in prostatic needle biopsies should be routinely examined with additional sections, particularly when immunohistochemical analysis is not possible. Some biopsies with atypical glandular proliferation may show focal carcinoma in additional sections, even if the immunohistochemical analysis did not provide a diagnosis of malignancy. Additional sections can also reveal areas of carcinoma that were not apparent in the original sections.

## 1. Background

Focal glandular prostatic atypia (FGA), also termed atypical small acinar proliferation (ASAP), has received a great deal attention, as it is often impossible to classify it as malignant or benign by needle biopsies (1, 2). Due to a lack of sufficient architectural criteria, cytological and immunohistochemical examinations often require additional biopsies for a conclusive diagnosis. The predictive value of biopsies with focal glandular atypia to carcinoma varies from 40% to 50% (1-5).

Although there have been numerous studies on morphological and immunohistochemical analysis of needle biopsies with ASAP, only a few studies have investigated the utility of examining additional sections in prostate biopsies (6, 7). Routine examination of additional histological sections in biopsies with ASAP may facilitate diagnosis, since the number of prostatic glands can markedly vary from one level to another (8). In 2 retrospective reviews (9, 10), we found that examining additional sections allowed the classification of focal glandular atypia in focal prostatic carcinomas in 18 of 63 biopsies (29%).

## 2. Objectives

The purpose of this review is to compare the diagnostic value of examining additional sections and immunohistochemical analysis in 30 biopsies with an original diagnosis of focal glandular atypia.

## 3. Patients and Methods

Thirty patients with ASAP examined in 2010 and 2012 were selected. Their age, findings on a digital rectal examination, and the levels of prostate-specific antigen at the time of the biopsy were recorded for all cases. The biopsies were performed on the basis of increased prostate-specific antigen or abnormal findings on digital rectal examination. All cases originally had a single slide with 3-micron sections. After the diagnosis of focal glandular atypia, immunohistochemical analysis using an antibody against high-molecular-weight keratin (1:50 dilution, 34BetaE12; Dako), and 2 additional slides with 3 sections each were analyzed. In total, each case of focal glandular was examined using 1 slide for immunohistochemistry (level one) and 6 additional sections (levels 2 and 3). In all cases, the following histological changes were recorded in the additional sections: (A) immunohistochemical result (helpful or not helpful); (B) increase or decrease in the number of atypical glands in subsequent sections; (C) increase or decrease of the following changes: infiltrative appearance, nucleomegaly, hyperchromatic nuclei, prominent nucleoli, apparent, basophilic or eosinophilic intraluminal secretions, crystalloids, mitosis, glomeruloid bodies, prostatic intraepithelial neoplasia, collagenous micronodules, and neural infiltration; (D) areas with carcinoma not seen in the original sections. The usefulness of additional sections in ASAP previously reported in the literature was recorded (7, 9, 10).

## 4. Results

The clinical and laboratory data are summarized in Box. The patients’ ages ranged from 47 to 82 years (mean, 69 years). In 24 cases, abnormalities were found in the digital rectal examination, including increased volume of the gland and/or induration of one or both prostatic lobes, suggesting carcinoma. In the remaining 10 cases, the prostate was normal or slightly increased in volume as ascertained by digital rectal examination. The prostate-specific antigen level ranged from 4.8 to 27 ng/mL (mean, 14.8 ng/mL). No patient had evidence of systemic disease or metastasis as determined by clinical or radiological studies.

**Table:** 

Box.	
Age of the patients	47 to 82 y (average: 68 y)
Abnormal digital rectal examination	24 patients
Prostate-specific antigen	4.8-27.5 ng/mL (mean: 14.8 ng/mL)

Table 1 summarizes the utility of additional sections in ASAP in the current study and in previous reports. Table 2 shows the value of additional sections and immunohistochemical analysis of 30 biopsies in the present study. In 4 cases, focal carcinoma was diagnosed using only the additional sections (13.3%). In 2 of these cases, focal areas with carcinoma that were not present in the original sections were observed. In 4 other cases, immunohistochemical analysis was the only useful method for diagnosing cancer. In 9 cases (30%), both methods were useful for classifying focal glandular atypia as carcinoma. However, in 6 biopsies, the criteria for carcinoma were more apparent in the additional sections. Thus, when all methods were used for diagnosis, including additional sections, immunohistochemistry, and a combination of both, 17 biopsies with focal glandular atypia (56.6%) were diagnosed as focal carcinomas.

**Table 1:** Additional Sections of ASAP a and Prostatic Carcinoma

**ASAP Biopsies, No.**	**Prostatic Carcinomas Biopsies, No.**	%
100 (7) ^b^	10	10
25 (9) ^b^	9	36
38 (10) ^b^	9	22.5
30 (current study)	10	33.3
Total (n = 193)	32	25

^a^Abbreviation: ASAP, atypical small acinar proliferation

^b^References

**Table 2:** Additional Sections vs. Immunohistochemical Study in Needle Prostate Biopsy

**Biopsies (n = 30)**	**Focal Carcinoma, No. (%)**
Additional levels	4 (13.3)
Immunohistochemical study a	4 (13.3)
Both useful (levels and IHC)	9 (30.0 )
Not useful (levels and IHC)	13 (43.4)
Total	30 (100)

^a^High-molecular-weight keratin (34BetaE12).

**Figure 1:**
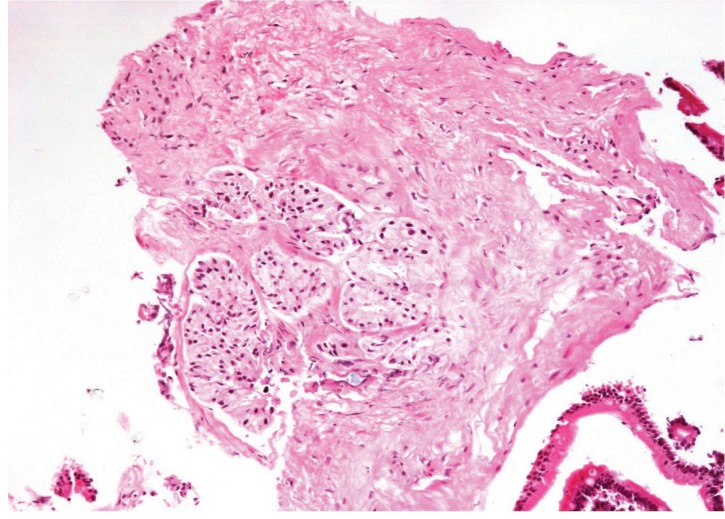
Atypical Small Acinar Proliferation in Prostatic Needle Biopsy. Nest of glands with scant atypia. Although the section is compatible with carcinoma, it is not possible to exclude complete prostatic adenosis.

**Figure 2:**
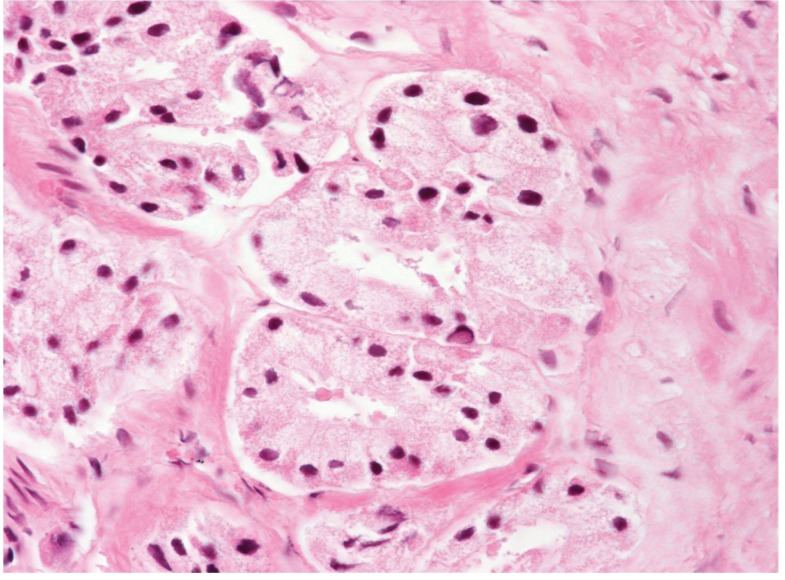
The Glands Show Clear Cytoplasm and Hyperchromatic Nuclei.

**Figure 3:**
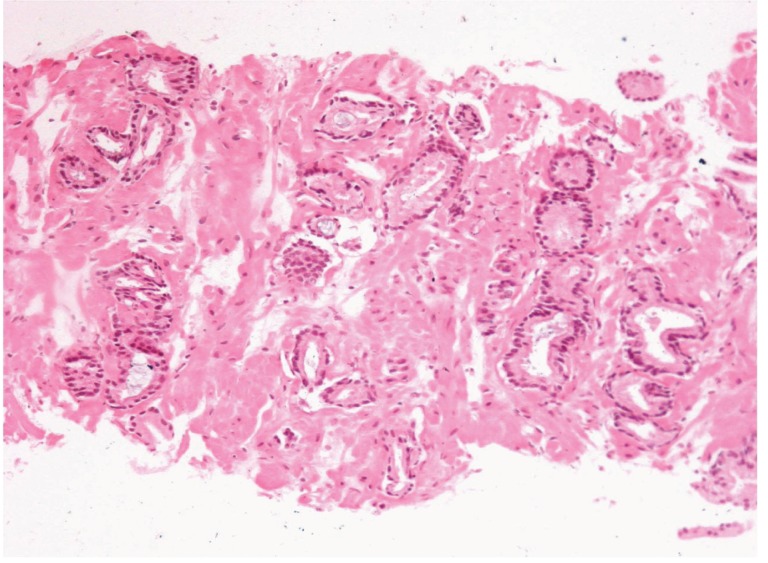
Additional Level (number Three). The Glands Show an Uneven Distribution and Infiltrative Pattern.

**Figure 4:**
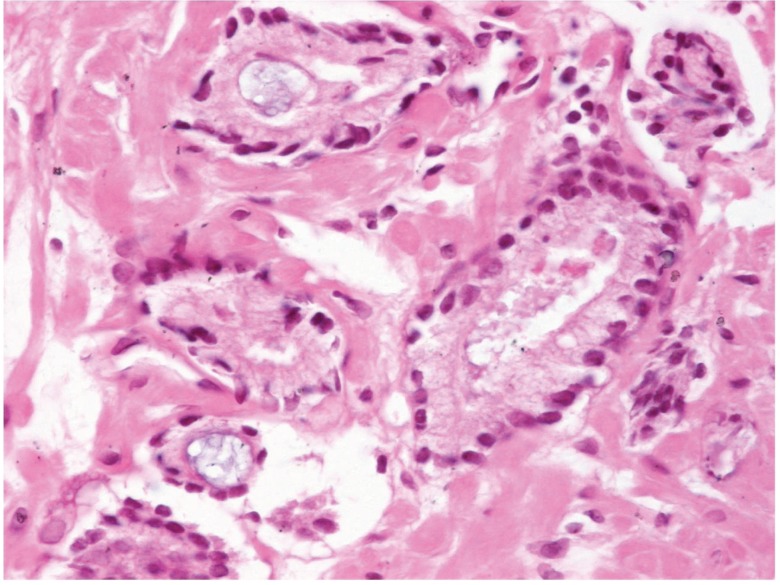
Eosinophilic and Basophilic Secretions in Neoplastic Glands.

Additional sections revealed several histological criteria for carcinoma in one-third of the biopsies. The most frequent finding included malignant glands with an increased infiltrative pattern, nucleomegaly, hyperchromatic nuclei, nucleoli apparent, rigidity of glandular lumen, and intraluminal basophilic or eosinophilic secretions (Figures 1,2, 3 and 4). The combination of architectural and cytological findings that enabled a diagnosis of carcinoma varied from one case to another. Thus, in some cases, there was an increased number of neoplastic glands with a clearly infiltrative pattern that was not apparent in the original cuts. These cases might or might not show cytological alterations, such as nucleomegaly, apparent nucleoli, rigidity of the glandular lumen, or intraluminal secretions. In other biopsies, the number of glands was similar in the additional sections compared to the original sections, but there was more apparent nucleomegaly and/or more prominent nucleoli, thereby facilitating a malignant diagnosis. Half of the cases had basophilic or eosinophilic intraluminal secretions, and 2 cases had crystalloids. Prostatic intraepithelial neoplasia, glomeruloid bodies, collagenous micronodules, or neural infiltration were not observed in the additional sections in any of the cases.

Interestingly, in 4 of the 30 biopsies, the diagnosis of carcinoma was established only by additional sections, as the immunohistochemical analysis did not demonstrate an absence of basal cells. Two of these biopsies showed other areas with malignant glands that were not evident in the original sections. These results emphasize the importance of analyzing additional sections in addition to immunohistochemical analysis. In practice, it will be important for the additional levels to be continuous with the ASAP area to prevent tissue loss. As such, communication between the pathologist and the laboratory technician is valuable. 

## 5. Discussion

The diagnostic utility of analyzing additional sections in prostate biopsies with ASAP has received little attention in the literature, despite being a simple and inexpensive method. Renshow et al. (6) reported that prostatic carcinoma is increasingly diagnosed by small foci of core needle biopsy specimens. However, the criteria for adequate sampling of this tissue have not been defined. In their study, 229 cases were reviewed, including 47 core specimens with ASAP and 22 with high-grade prostatic intraepithelial neoplasia. Six core specimens were obtained for each case, and 2 blocks were prepared containing 3 cores each. Three slides containing multiple serial sections were prepared from each block. They established that 13% of atypical foci and 3% of carcinomatous foci were not present on the first slide of the prostate core needle biopsy specimens, because the tissue containing these foci was not cut. This report indicated that at least 3 slides are necessary in order to avoid or reduce false negative results (6).

Reyes and Humphrey (7) recognized that no data have been published on the diagnostic yield of complete histopathologic examination of prostate needle biopsy specimens. Therefore, they evaluated the diagnostic utility of obtaining additional sections after a non-malignant diagnosis was given on 3 initial slides. This prospective study included 200 consecutive prostatic biopsies. They compared 100 cases originally diagnosed with atypia (including high-grade prostatic and intraepithelial neoplasia and ASAP) to those of 100 cases originally diagnosed as benign. They performed complete histologic analysis of the prostate needle biopsy specimens with serial sections from both groups. In 4 of the 40 cases with focal glandular atypia (10%), definitive carcinoma was present on additional sections, including the first additional slide. No case with an original diagnosis of benign prostatic tissue (n = 100) or high-grade PIN (n = 60) were diagnosed with carcinoma diagnosed on additional slides. As such, the authors recommended obtaining a single additional slide with several 3-micron sections after a diagnosis of focal glandular atypia on the bases of 3 initial slides of prostatic needle biopsy.

In our first study, we found that additional sections were useful for classifying ASAP as carcinomas in 9 of 25 (36%) biopsies (9). The utility was closely associated with increased number of glands observed, which increased from an average of 10 to 17 glands in the affected area. This increase in glands facilitated recognition of an infiltrative appearance of the glands. Several other criteria for the diagnosis of carcinoma became apparent, including prominent nucleoli, intraluminal secretions, rigid glandular lumen, glandular fusion, and glomeruloid bodies. In a second study (10), we included 38 cases in which prostate needle biopsies were performed and showed focal glandular atypia in one isolated microscopic field. Each case originally had 3 sections of 3 microns per slide. In all cases, 3 additional slides were made with 3 additional sections. In total, each case had 12 sections (the original 3 and an additional 9). The additional sections enabled a definitive diagnosis of malignancy in 9 of the 38 cases (22.5%). In the other 29 biopsies, the area with glandular atypia was less apparent or disappeared altogether.

In the current study, we found that additional sections revealed several architectural and cytological histological criteria for carcinoma in one-third of the biopsies. In 4 of the 30 biopsies, the diagnosis of carcinoma was established only by additional sections, as the immunohistochemical analysis revealed no convincing absence of basal cells. Two of these biopsies showed other areas with malignant glands that were not apparent in the original sections. These results emphasize the importance of performing immunohistochemistry simultaneously with analyzing additional sections. The differences between results from previous studies (7, 9, 10) and the present study may be related to the selection criteria for the diagnosis of ASAP. In our 3 studies, all the cases showed architectural and cytological features highly suggestive of carcinoma. In addition, the convenience of extended sampling at the first biopsy appears to be useful for detecting a greater number of prostatic carcinomas (11).

The utility of additional sections for diagnosing other prostatic diseases, such as precursor lesions of prostate cancer, focal atrophy, post-atrophic hyperplasia, and atypical adenomatous hyperplasia (adenosis) (12), will require further studies. Nevertheless, it is probable that additional sections may facilitate the diagnosis of prostatic lesions that are difficult to interpret. High-molecular-weight keratin was negative in areas of ASAP in 13 cases, supporting the diagnosis of carcinoma. In 4 other biopsies, the immunohistochemical analysis was not assessed, because the suspicious area could not be found in the sections or for technical reasons. It is well demonstrated that 34BE12 is highly susceptible to the effects of formalin fixation. Varma et al. (13) studied the deleterious effects of prolonged formalin fixation and concluded that 34BE12 immunoreactivity is dependent on optimal fixation.

In conclusion, the reviewed studies demonstrate that analyzing additional sections is useful in approximately 25% of biopsies with ASAP. Making additional cuts in biopsies with focal glandular atypia is a fast and inexpensive method that can be routinely practiced, particularly if resources for immunohistochemical studies are not available. Some biopsies with ASAP may show other areas of carcinoma that were not apparent in the original sections. Recognizing these lesions is important, since many correspond to small and potentially curable tumors.
